# Night-Migratory Songbirds Possess a Magnetic Compass in Both Eyes

**DOI:** 10.1371/journal.pone.0043271

**Published:** 2012-09-12

**Authors:** Svenja Engels, Christine Maira Hein, Nele Lefeldt, Helmut Prior, Henrik Mouritsen

**Affiliations:** 1 AG “Neurosensorik/Animal Navigation”, IBU & Research Center for Neurosensory Sciences, Carl-von-Ossietzky-University of Oldenburg, Oldenburg, Germany; 2 Institute of Psychology, Goethe-University, Frankfurt/Main, Germany; Pennsylvania State University, United States of America

## Abstract

Previous studies on European robins, *Erithacus rubecula*, and Australian silvereyes, *Zosterops lateralis*, had suggested that magnetic compass information is being processed only in the right eye and left brain hemisphere of migratory birds. However, recently it was demonstrated that both garden warblers, *Sylvia borin*, and European robins have a magnetic compass in both eyes. These results raise the question if the strong lateralization effect observed in earlier experiments might have arisen from artifacts or from differences in experimental conditions rather than reflecting a true all-or-none lateralization of the magnetic compass in European robins. Here we show that (1) European robins having only their left eye open can orient in their seasonally appropriate direction both during autumn and spring, i.e. there are no strong lateralization differences between the outward journey and the way home, that (2) their directional choices are based on the standard inclination compass as they are turned 180° when the inclination is reversed, and that (3) the capability to use the magnetic compass does not depend on monocular learning or intraocular transfer as it is already present in the first tests of the birds with only one eye open.

## Introduction

Each year, migratory birds travel long distances between their breeding grounds and their wintering quarters, and it is well established that they use a light-dependent magnetic compass for orientation [1]–[11]. The direction of the Earth's magnetic field is supposedly sensed by radical pair-forming, light-dependent photopigments in the birdś eyes [4], [11]–[20] and then processed in Cluster N, a specialized, night-time active, light-processing forebrain region [9], [21]–[24] which is required for magnetic compass orientation [10].

In 2002, Wiltschko and colleagues published data on European robins, *Erithacus rubecula*, suggesting that these birds are unable to orient with the help of the geomagnetic field when using their left eye only [25]. Subsequently, Wiltschko et al. [26] reported a similar all-or-none lateralization of magnetic compass orientation in favor of the right eye and left brain hemisphere in a diurnally migrating songbird, the Australian Silvereye, *Zosterops lateralis*. These findings have led to the notion that the vision-mediated magnetic compass is located only in the right eye of migratory birds, whereas input from the left eye only is not sufficient for magnetic compass orientation [25]–[28]. A complete left hemispheric (right eye) lateralization of the magnetic compass would, however, be at variance with what has been found so far for vision-based orientation in birds. While slight to moderate lateralization is commonly found during visually guided tasks, except for the two earlier findings on robins and silvereyes, no other functions involving the visual system have been shown to be lateralized in an all-or-none fashion (e.g. [29]–[31]). A strong lateralization would also seem counterproductive from an evolutionary perspective. The survival of a bird having a magnetic compass located exclusively in its right eye would be more easily affected by eye-infection or monocular damage than a bird having a functional magnetic compass in both eyes. Likewise, the possibility that birds may show uni-hemispheric sleep during flight [32] would favor bilateral perception of magnetic compass directions in a night-migratory bird.

In addition, functional neuroanatomical data have questioned the lateralization of the magnetic compass towards the right eye: cryptochromes, the most promising candidates for primary sensory molecules involved in the radical pair mechanism are found in both eyes with no obvious difference in cryptochrome expression or connectivity during a magnetic compass orientation task [9], [16]. Cluster N, which has been shown to be involved in the magnetic compass information processing circuit [10], [11], [21], is active in both brain hemispheres of European Robins and garden warblers, *Sylvia borin*, when performing magnetic compass guided orientation [21]–[23]. In fact, neuronal activation patterns in Cluster N of European Robins were slightly but significantly lateralized towards the right brain hemisphere, i.e. in the opposite direction to the one suggested by Wiltschko and colleagues [22]. A quantification of neuronal activity revealed a dominance of the right brain hemisphere, which – due to the almost complete crossover of the fibers of the optic nerve of birds (e.g. [33], [34]) – gets its input mainly from the left eye [22]. Furthermore, a study on the magnetic compass performance of garden warblers [35], another night-migratory songbird species, and in Pekin ducks [36], found no lateralization effect. The birds were able to orient with both eyes open, with the left eye open only, and with the right eye open only.

**Figure 1:**
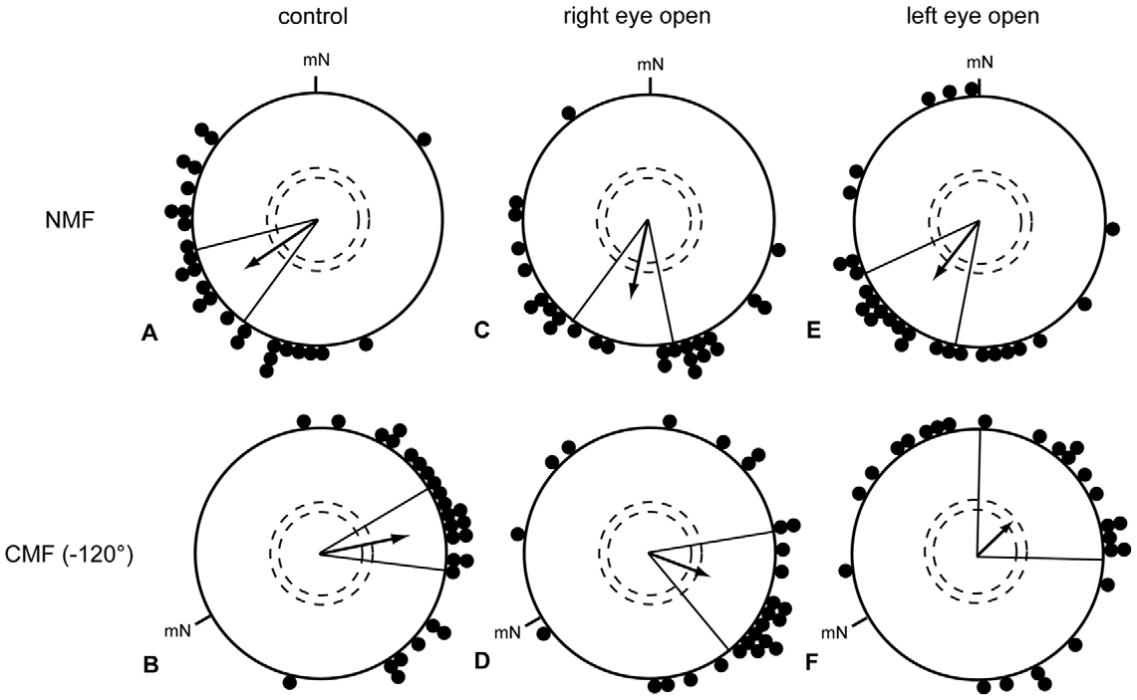
Birds can use their magnetic compass if light and/or visual input reaches any one eye. A–B: European robins equipped with eye covers with a hole in front of both eyes, C–D: birds equipped with eye covers allowing light and visual input to reach only the right eye, E–F: birds equipped with eye covers allowing light and visual input to reach only the left eye. The data in A, C, and E were collected in an unchanged magnetic field (NMF). The data in B, D, and F were collected in a magnetic field turned 120° counter clockwise (CMF). mN  =  magnetic North. For description of the circular diagrams, see legend to Fig. 2 (redrawn after Hein et al. [41]).

To exclude the possibility that species-related differences between garden warblers and European robins had caused the contradictory results, we independently repeated the experiments of Wiltschko et al. [25] using the same species, namely European robins. We had tested them during autumn migration when they use simple compass orientation [37]–[40]. The European Robins were also able to orient using their magnetic compass with both eyes open, the right eye open only or the left eye open only ([41], Fig. 1).

When these new robin data [41] were published as a commentary to Wiltschko et al. [25] the original authors suggested three possible explanations for the contradictory results [42] (1) There may be seasonal differences in the ability to orient with the left eye since we tested the birds during autumn migration [35], [41] whereas Wiltschko et al. [25] tested them during spring migration. The rationale behind this explanation relates to the fact that the birds might to a higher degree rely on learned map-based information on their way home in spring than on their way out in autumn [37]–[40], [43]. As strong lateralization of (olfactory) map-based orientation has been reported [44], [45] the putative seasonal differences in Wiltschko et al. [25] could reflect a lateralization of a navigational map rather than a lateralization in peripheral sensory processing. (2) In Hein et al. [41] birds did not use their magnetic compass but showed some kind of ‘fixed direction response’ [46] (3) The higher total number of tests per bird carried out by Hein et al. [41] led to an interhemispheric transfer [47] which might mask the normal pattern of lateralization.

The aim of the present paper was to address all of these three questions in order to clarify whether the capability of magnetic compass orientation with both brain hemispheres is likely to represent the natural pattern or whether the differences might merely reflect differences in experimental procedures.

## Materials and Methods

### Magnetic fields

Magnetic fields were produced with double-wrapped, three-dimensional Merritt four-coil systems [48] with average coil diameters of about two meters. All experiments were performed within the central space of the coils where the heterogeneity was <1% of the applied field. Before the beginning of each experiment, the ambient magnetic field was measured in the centre and at the edges of the experimental volume within which the orientation cages were placed. Birds were tested in three different magnetic conditions: in a magnetic field resembling the natural one of Oldenburg (Natural Magnetic Field, NMF: MF strength  = 48,900 nT±150 nT [s.d.]; inclination  = 67.7°±0.6°; horizontal direction  = 360°±0.1°), in a magnetic field turned 120° counter-clockwise (Changed Magnetic Field, CMF: MF strength  = 49,000 nT±470 nT; inclination  = 68.0°±1.1°; horizontal direction  = −120°±0.5°) and in a magnetic field with reversed inclination (Inverted Magnetic Field, IMF: MF strength  = 48,110 nT±460 nT; inclination  = −67.5±0.5°; horizontal direction  = −0.2±1.3°). To produce the CMF and IMF condition, the current ran through the two subsets of windings of the four-coil system in the same direction. Under the NMF condition, the same current that we used to produce the CMF condition ran through the two subsets of windings but in opposite directions so that no significant changes (i.e. <10 nT) to the magnetic field were produced by the coils.

### Test subjects

In our study, we tested a total of 55 European robins. The birds were caught on the campus of the University of Oldenburg, Germany. The birds were housed indoors in individual cages in a windowless room under a light regime simulating the local photoperiod. The behavioral experiments were performed during the autumn migratory seasons in 2009 and 2010 and during the spring migratory season 2011 on the campus of the University of Oldenburg. All animal procedures were performed in accordance to the local and national guidelines for the use of animals in research and approved by the Animal Care and Use Committees of the Niedersächsisches Landesamt für Verbraucherschutz und Lebensmittelsicherheit (LAVES), Oldenburg, Germany.

### Behavioural experiments

The birds were tested in orientation cages inside wooden huts placed on the university campus, where no other cue than the geomagnetic field was available. The walls and ceilings of the huts were lined with grounded aluminum shields, which acted as Faraday cages and shielded non-stationary electromagnetic disturbances by approximately two orders of magnitude. All power supplies and other equipment were placed in a separate room in a shelf that was also shielded by aluminum to minimize electromagnetic disturbances.

One hour (±10 min) before the experiments started (0–30 min after sunset), the birds were placed outdoors in wooden transport cages that allowed them to see parts of the evening sky to give them the possibility to calibrate their magnetic compass from twilight cues [49]–[52]. Immediately thereafter, they were placed in modified aluminum Emlen funnels (35 cm diameter, 15 cm high, walls 45° inclined; [53]), which were coated with scratch sensitive paper [54] on which the birds left scratches as they moved. The sequence of testing conditions (NMF or CMF as well as right eye open, left eye open or both eyes open) varied between the individual birds, partly randomly, partly depending on the current number of active and oriented tests per condition. The IMF and spring tests were done exclusively with birds having their left eye open only. Furthermore, the specific funnel position (9 funnels per hut) and/or hut in which a bird was tested were alternated between test rounds and between nights, so that no consistent local room or funnel cues could putatively be remembered by the birds and putatively transferred between tests and conditions. Thus, due to these procedures, it can be completely excluded that birds with their left eye open only could have oriented because they had calibrated the testing huts or directional cues from the surrounding environment when they were tested with both eyes open.

The overlap point of the paper was adjusted to one of the cardinal directions (N, S, E or W). This overlap point was changed randomly between huts and nights. This is important because the papers are always evaluated relative to the overlap point. When the evaluators of the papers do not know if the overlap point was in N, S, E or W, it becomes impossible for “wishful thinking” to influence the results, since one cannot know which geographical direction is equivalent to a certain direction on the paper. The location of the overlap point is only revealed and taken into consideration, after the primary evaluation of the papers has taken place.

The birds were tested for one hour under dim light conditions (2.1 mW/m^2^) produced by incandescent bulbs (spectrum in [10]). In each hut, nine birds were tested simultaneously. A second test of a given night started 1.5 hours (±10 min) after the first one, and each bird was tested in a different hut compared to the first test that night but under the same magnetic field condition (NMF, CMF or IMF). In spring 2011, occasional third tests were started when the birds had been highly active during the second round of tests. The orientation directions of the first, second and third test can therefore be treated as independent and thus were all entered into the calculation of the mean direction of each individual bird. The magnetic field conditions applied in a given hut were switched approximately every second night, and usually two different magnetic field conditions were tested in different huts on any given night.

Before the eye cover experiments started, we tested the birds without wearing eye covers for several nights to ensure that they were in migratory mood and to get a control direction. For the eye cover experiments, we used the same procedures as in the control experiments, except that the birds were fitted with eye covers just before they were placed outdoors for one hour in the wooden transport cages. The eye covers (<0.5 g) were sewed of light-tight, artificial leather with tightly fitted openings left for the beak and the neck. In addition, they had openings of 8 mm diameter in front of both eyes (controls), the right eye only, or the left eye only. The eye covers reduced the ambient light by at least five orders of magnitude, which means that the light intensity under the hoods during the experiments was <1*10^−5^ mW/m^2^, and neuronal activity of Cluster N was reduced to background level by the eye covers [35]. The eye covers were removed every night immediately after the end of the behavioral tests. This technique of covering the eyes differed from the one used in [25]. We considered our eye covers preferable because comparative control tests had shown that the technique used in the present study was much less stressful for the birds.

### Orientation data analysis

Two researchers visually determined each bird's mean direction from the distribution of the scratches independently from each other [55]. The evaluation of the papers were blinded, i.e. the evaluators did not know the direction of the overlap point of the paper (see above) nor the magnetic field condition experienced by the bird. If the two researchers considered the scratches to be randomly distributed or if the two independently determined mean directions deviated by more than 30°, a third independent researcher was asked to determine the mean direction. If this third individual determined a mean direction similar to one of the first two, and if the individual with initially differing opinion also agreed with this direction, the mean of the two similar directions was recorded as the orientation result. If the three independent researchers could not agree on one mean direction, the bird's heading was defined as random and excluded from the analyses (17% of all control tests; 12% of all eye cover tests). Birds with fewer than 100 scratches on the paper were considered inactive and were also excluded from the analysis (35% of all control tests; 41% of all eye cover tests). The currently used thermal paper [54] is much more scratch sensitive than the previously used type writer correction paper. We observed that birds placed in funnels and removed immediately afterwards already left up to 80 scratches on the paper while they initially try to escape in random directions. Therefore, we required a minimum of 100 scratches for inclusion of a test in further analyses compared to the limit of 30 scratches used in the past (e.g. [10], [35]). The average mean heading for each bird was calculated from all its oriented tests recorded under a given experimental condition. Based on these individual mean vectors, group mean vectors were calculated and the significance of the group mean vector was tested using the Rayleigh-test [56]. The Mardia-Watson-Wheeler-test (MWW) was used to test for differences in the mean orientation between the different magnetic field conditions [56].

## Results

**Figure 2:**
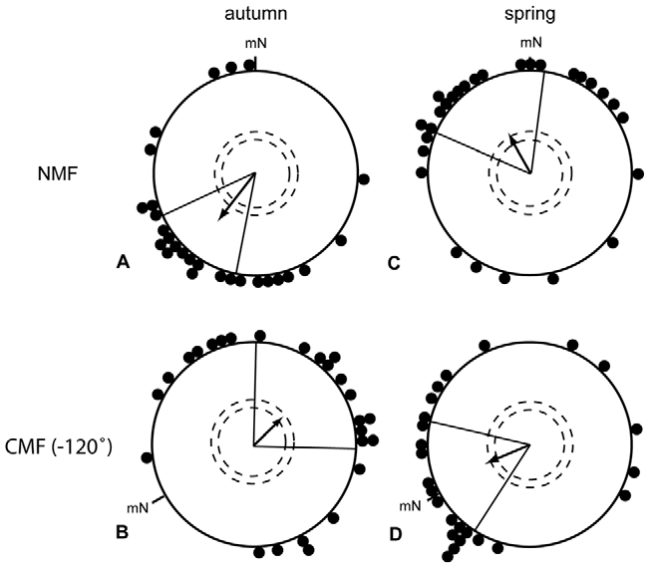
Birds can use their magnetic compass in autumn and spring using only their left eye. European robins equipped with eye covers allowing light and visual input to reach only the left eye were tested in autumn (A, B) and spring (C, D). The data in A and C were collected in an unchanged magnetic field (NMF). The data in B and D were collected in a magnetic field turned 120° counter clockwise (CMF). A, B are redrawn after Hein et al. [41]. mN  =  magnetic North. The arrows indicate the group mean vectors. The inner and outer dashed circles indicate the radius of the group mean vector needed for significance according to the Rayleigh Test (p<0.05 and p<0.01 respectively). The lines flanking the group mean vector indicate the 95% confidence intervals for the group mean direction.

In Hein et al. [41], we showed that European robins equipped with monocular eye covers that enabled them to see with their left eye only were significantly oriented into their appropriate migratory direction under the NMF condition (217°±27°, r = 0.57, p<0.001, N = 27; Fig. 2A), as well as under the CMF condition (47°±45°, r = 0.38, p<0.05, N = 26; Fig. 2B). The easterly direction during the CMF condition was not significantly different from the expected migratory direction towards approximately 85°–95° (ca. 210°–120°, see [40]), because the expected migratory direction was within the 95% confidence interval of the group mean orientation direction (2°–92°). The mean orientation of the birds, which had only their left eye open, tested in the CMF condition differed significantly and in the expected direction from the same birds' orientation in the NMF condition (95% confidence intervals do not overlap; MWW: W = 22.76, p<0.001).

In order to test whether the experimental season might have an influence on the degree of lateralization of the magnetic compass, we tested 28 birds with light reaching their left eye only during the spring migratory period. Due to the limited amount of experiments that can be performed during one migratory season, we restricted our tests on the left eye open condition, because this was the eye cover condition under which Wiltschko et al. [25] had found no magnetic compass orientation. European Robins that had only their left eye open oriented in their expected spring migratory direction towards north-east in the unchanged magnetic field (NMF: 331°±37°, r = 0.42, p<0.01, N = 28; Fig. 2C) and changed their orientation according to the 120° turn counter-clockwise (CMF: 246°±35°, r = 0.47, p<0.01, N = 25; Fig. 2D). The mean orientation in the CMF condition differed significantly and in the expected direction from the same birds' orientation in the NMF condition (95% confidence intervals do not overlap; MWW: W = 8.52, p<0.05).

**Figure 3:**
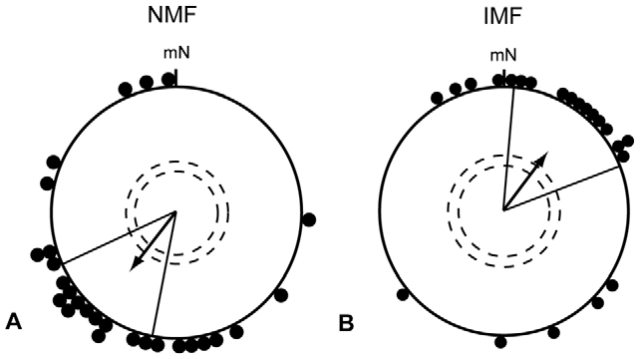
Birds that could only use their left eye reversed their orientation according to the inclination. Results from European robins equipped with eye covers allowing light and visual input to reach only the left eye. The data in A were collected in an unchanged magnetic field (NMF), and are redrawn after Hein et al. [41]. The data in B were collected in a magnetic field with an inverted vertical component (IMF). mN  =  magnetic North. For description of the circular diagrams, see legend to Fig. 2.

To ensure that our birds used their inclination compass, we repeated the monocular left eye condition in autumn in a magnetic field where the vertical component was reversed. The birds reversed their orientation as expected when they use an inclination compass (IMF: 37°±33°, r = 0.55, p<0.001, N = 22; Fig. 3B). The mean orientation in the IMF condition differed significantly from the NMF condition in Hein et al. [41] (NMF: 217°±27°, r = 0.57, p<0.001, N = 27; Fig. 3A; 95% confidence intervals do not overlap; MWW: W = 23.15, p<0.001).

While Wiltschko et al. [25] tested each bird only twice, we did more tests to reduce noise in the data and to make sure that our results are consistent to internal replication. However, because Wiltschko et al. [42] suggested that the different number of tests might be the reason for the difference in the orientation results, we also reanalyzed our data reported in Hein et al. [41] from the 27 birds tested in autumn based only on the first two oriented (i.e. not random) and active tests of each bird in each of the six experimental conditions (see Table 1). There, too, the orientation was significant in all test conditions except of the “right eye open condition” in the changed magnetic field (but this condition also showed a clear tendency in the expected direction). In conclusion, the capability to orient with both eyes, the left eye only, or the right eye only was present from the very beginning. Thus, the differing number of tests conducted per bird between our study [41] and that of the Wiltschkos [25] cannot be the cause for the differing results.

**Table 1:** Autumn orientation results if only the first two active and oriented tests per bird are considered.

	Control/both eyes open			Right eye open			Left eye open		
	α	N	r	α	N	R	α	N	r
NMF	241	27	0.447**	180	27	0.349^ns^	235	27	0.490**
CMF	47	25	0.441**	109	27	0.291^ns^	27	26	0.368*

NMF  =  normal magnetic field; CMF  =  changed magnetic field; α  =  group mean direction; N  =  number of individuals; r  =  length of the mean vector; ns not significant; * p<0.05; ** p<0.01, significance by the Rayleigh test.

**Figure 4:**
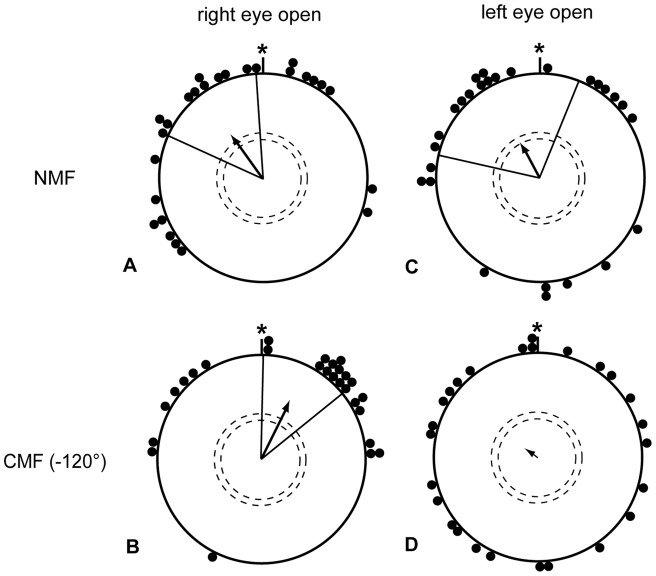
Within-subject comparisons of the monocular orientation of European robins. Comparison of each of the monocular conditions with the binocular mean of each individual taken as a reference (i.e. taken as zero value, *). There is no systematic difference between the angular deviations of the right eye open condition (A 325°±32°, r = 0.51, p<0.001, N = 26; B 27°±26°, r = 0.60, p<0.001, N = 27) and the left eye open condition (C 333°±51°, r = 0.35, p<0.05, N = 27; D 310°, r = 0.13, p = 0.65, N = 26) in neither the normal magnetic field condition (NMF A, C), nor in the changed magnetic field condition (CMF B, D) For description of the circular diagrams, see legend to Fig. 2.

**Figure 5:**
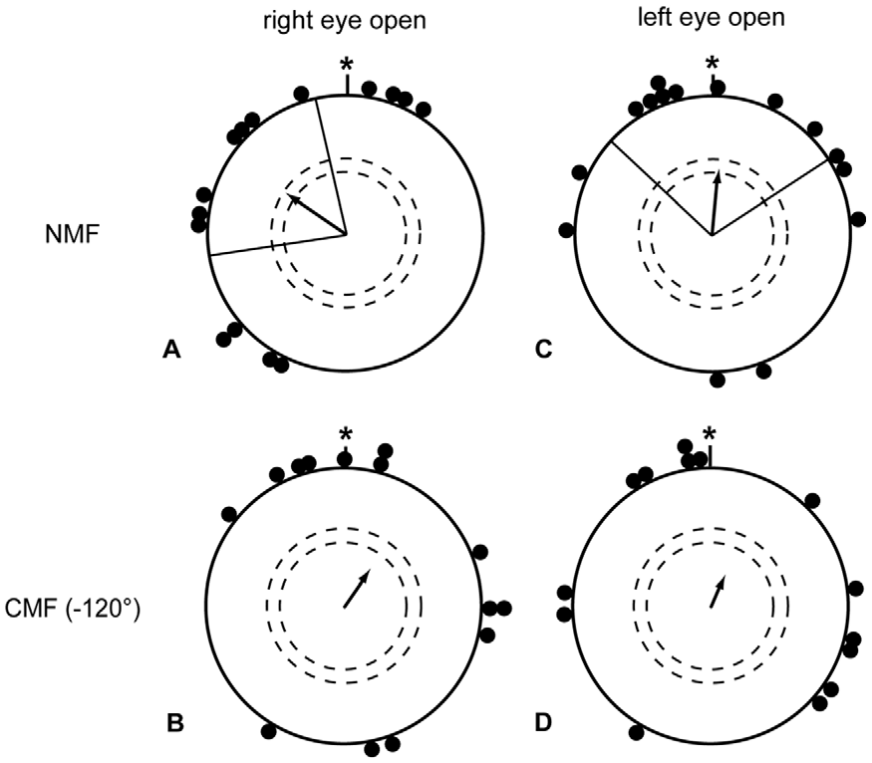
Within-subject comparisons of the monocular orientation of garden warblers. Comparison of each of the monocular conditions with the binocular mean of each individual taken as a reference (i.e. taken as zero value, *). There is no systematic difference between the angular deviations of the right eye open condition (A 304°±44°, r = 0.51, p<0.05, N = 15; B 35°, r = 0.32, p = 0.24, N = 14) and the left eye open condition (C 4°±52°, r = 0.45, p<0.05, N = 15; D 25°, r = 0.23, p = 0.48, N = 14) in neither the normal magnetic field condition (NMF A, C), nor in the changed magnetic field condition (CMF, B, D). For description of the circular diagrams, see legend to Fig. 2. Data from Hein et al. [35].

In all studies with monocular occlusion on orientation in pigeons carried out so far, there was a strong and reliable bias into the direction of the open eye in monocular birds (e.g. [57]–[60]). The orientation of individuals with the right eye open deviated markedly in a clockwise direction and the orientation of individuals with the left eye open deviated in a counterclockwise direction as compared to binocular controls. As this systematic bias might indicate how information from either eye is integrated in the brain (c.f. discussion), we analyzed whether such a bias also occurs in eye-capped songbirds performing magnetic compass orientation in Emlen funnels. For each individual, the angular deviation with the left or right eye from the binocular mean of the same individual was calculated (comparing the orientation of each individual bird from Fig. 1C and 1E with the same individual birds' orientation in Fig. 1A and by comparing the orientation of each individual bird from Fig. 1D and 1F with the same birds' orientation in Fig. 1B). There was no significant difference between the angular deviations with the left and right eye in any of the two magnetic field conditions (Fig. 4; NMF condition: MWW n.s.; CMF condition: no test possible because one distribution is random). A similar analysis of the orientation of garden warblers, which have also been shown to be able to use their magnetic compass with the left as well as with the right eye [35], revealed the same pattern (Fig. 5).

Here, it is important to stress that the lack of significance in Fig. 4D is not suggesting that the left eye open birds could not orient in the CMF condition, since the basic directional choices of the same individual birds (shown in Fig. 1F) show significance. In Figure 4D, we only look at the relative orientation of the same individuals with both eyes open and with the left eye open in the CMF condition to test for systematic side biases. The apparent “disorientation” in Fig. 4D just indicates a quite strong variability between the direction chosen in the both eyes open condition and the left eye open only condition. We believe this to be a statistical coincidence that must happen once in a while, when doing many experiments. This view is supported by the fact that the garden warblers show almost identical relative distribution when the left-eye open only and the right eye open only conditions are compared with the same individual birds' orientation in the both eyes open condition (compare Fig. 5A with 5C, and Fig. 5B with 5D). Thus, during magnetic compass orientation, a bias towards the side of the open eye, which is found with typical visual cues, was absent.

In order to test whether migratory experience might have influenced the degree of lateralization of the magnetic compass, we tested the directional preferences of the six adult birds that took part in our experiments with the mean direction of the 21 juvenile birds as a reference (Table 2). If the formation of a learned navigational map would be a key component of the lateralization pattern found in earlier experiments [25], [26], one should expect that there is little or no difference in autumn migrants, but a difference in the strength of lateralization in spring migrants with the lateralization becoming stronger in older experienced birds. On average, the mean direction of the adult birds deviated by 22° from that of the juvenile birds, with a mean (NMF and CMF) of 37° deviation for the left eye open condition and a mean of 15° for the right eye open condition. The mean vector lengths of adult bird orientations were similar to those of the juvenile birds. Our data do not indicate that age/experience leads to a strong increase or decrease in lateralization of the magnetic compass in European Robins (Table 2), although it has to be considered that the number of subjects compared was low.

**Table 2:** Orientation results in autumn according to the age of the birds.

		Control/both eyes open			Right eye open			Left eye open		
		Α	N	r	α	N	R	α	N	r
**Juvenile**	NMF	234	21	0.720***	189	20	0.651***	216	21	0.548**
	CMF	75	21	0.742***	115	21	0.577***	69	20	0.388*
**Adult**	NMF	244	6	0.592^ns^	203	6	0.654^ns^	221	6	0.644^ns^
	CMF	91	6	0.657^ns^	96	6	0.342^ns^	360	6	0.645^ns^

NMF  =  normal magnetic field; CMF  =  changed magnetic field; α  =  group mean direction; N  =  number of individuals; r  =  length of the mean vector; ns not significant; * p<0.05; ** p<0.01; *** p<0.001, significance by the Rayleigh test.

## Discussion

The present study demonstrates that European robins equipped with eye covers allowing light and visual input to reach only the left eye can use their magnetic compass in autumn as well as in their spring migratory period and that this capability is present from the first tests onwards. Furthermore, by unequivocally demonstrating the use of an inclination compass we can rule out the possibility suggested by Wiltschko et al. [42] that the data of Hein et al. [41] were based on a ‘fixed direction’ response instead of true magnetic compass orientation. Thus, the present results confirm and extend the data presented in Hein et al. [41]. The magnetic compass of night-migratory birds is located in both eyes and not strongly lateralized. The lack of an all-or-non lateralization towards the right eye is also in agreement with numerous functional neuroanatomical data (for details [9], [10], [16], [21], [22], [35]; see introduction). Slight lateralization effects might, of course, still arise through hemispheric differences in higher level processing [31], [61], [62], such as for example a suggested preference for the processing of directional information of the left brain hemisphere in pigeons [31]. But these potential hemispheric differences would not result in an all-or-none lateralization.

The absence of a systematic bias towards the side of the open eye in the monocular conditions (Fig. 4) further supports the view that magnetic compass information is perceived independently with either eye. Orientation studies with pigeons in the field and in the laboratory revealed a strong and very reliable systematic bias towards the direction of the open eye [59], [60]. The origin of this bias is not fully understood yet, but the fact that it already takes place when the birds are sitting still before being released [58] makes a motor or turning bias unlikely and rather suggests a representational bias at the brain processing level. Such a representational bias would be likely to occur in a system where competing information from each side has to be integrated into a panoramic bilateral representation, which is then used to generate the output. If the input from only one side is sufficient for generating the normal behavioral output, removal of the input from the one or other side will not affect the overall balance of the system. Thus, the absence of a systematic angular deviation during magnetic compass orientation in songbirds suggests that each hemisphere can translate compass information from the contralateral eye into a valid migratory direction. Wiltschko et al. [25] also had suggested that a lateralization in favor of the right eye might be due to the magnetic stimulus being perceived like an object in combination with a left-hemispheric advantage for object-vision in birds. Absence of a lateralization under natural light conditions and lack of a directional bias as it is found during orientation in the presence of visual landmarks does not support the object-vision hypothesis.

The only remaining explanation for the differing results related to a lateralization of the magnetic compass is that they arose because of differences in the experimental paradigm. Lateralization of directional information might depend on environmental as well as on experimental conditions (e.g. [31]). One important difference is that all Oldenburg experiments were conducted double-blind while the Frankfurt experiments were not. The second major difference between the experimental procedures in Wiltschko et al. [25], [26] and Hein et al. [35], [41] was that the Frankfurt experiments were performed under unnatural green light, whereas the Oldenburg experiments were conducted under broad spectrum white light. The different light regimes might be important, particularly when one considers that, in recent years, many orientation responses of birds tested under different combinations and intensities of coloured light of rather narrow wavelength ranges have been reported that are difficult to explain. Maybe the result of Wiltschko et al. [25], [26] is another such example. It has been suggested that there might be lateralized differences in the distribution of colour-sensitive receptors in the retina of songbirds [63]. Although the functional significance of this lateralization is not fully understood, it might favour artefactual lateralization effects, which are absent under normal visual stimulation.

In conclusion, the notion of a strong right eye lateralization of the magnetic compass of migratory songbirds [25], [26] cannot be supported by double-blind, independent experiments performed in our lab. The data presented here, together with the results conducted with European robins [41], garden warblers [35], pigeons [62] and ducks [36], suggest that potentially all bird species can perceive and process magnetic compass information with any single eye if they are forced to do so. In other words, birds can use the right eye and left brain hemisphere as well as the left eye and right brain hemisphere for visual magnetic compass orientation. It is very possible that some smaller degree of lateralization of magnetic information processing exists in birds (e.g. [22], [31], [57]). However, our data show that the magnetic compass of night-migratory songbirds is not strongly lateralized and certainly not located in only one of the birds' eyes.
